# MPP+ induces necrostatin-1- and ferrostatin-1-sensitive necrotic death of neuronal SH-SY5Y cells

**DOI:** 10.1038/cddiscovery.2017.13

**Published:** 2017-02-27

**Authors:** Keisuke Ito, Yutaka Eguchi, Yusuke Imagawa, Shuji Akai, Hideki Mochizuki, Yoshihide Tsujimoto

**Affiliations:** 1 Laboratory of Molecular Genetics, Department of Medical Genetics, Osaka University Graduate School of Medicine, 2-2 Yamadaoka, Suita, Osaka 565-0871, Japan; 2 Department of Molecular and Cellular Biology, Research Institute of Osaka Medical Center for Cancer and Cardiovascular Diseases, 1-3-2 Nakamichi, Higashinari-ku, Osaka 537-8511, Japan; 3 Department of Neurology, Osaka University Graduate School of Medicine, Suita, Japan; 4 Laboratory of Synthetic Medicinal Chemistry, Osaka University Graduate School of Pharmaceutical Sciences, 1-6 Yamadaoka, Suita, Osaka 565-0871, Japan

## Abstract

Regulation of cell death is potentially a powerful treatment modality for intractable diseases such as neurodegenerative diseases. Although there have been many reports about the possible involvement of various types of cell death in neurodegenerative diseases, it is still unclear exactly how neurons die in patients with these diseases, thus treatment strategies based on cell death regulation have not been established yet. To obtain some insight into the mechanisms of cell death involved in neurodegenerative diseases, we studied the effect of 1-methyl-4-phenylpyridinium (MPP+) on the human neuroblastoma cell line SH-SY5Y (a widely used model of Parkinson’s disease). We found that MPP+ predominantly induced non-apoptotic death of neuronally differentiated SH-SY5Y cells. This cell death was strongly inhibited by necrostatin-1 (Nec-1), a necroptosis inhibitor, and by an indole-containing compound (3,3′-diindolylmethane: DIM). However, it occurred independently of receptor-interacting serine/threonine-protein kinase 1/3 (RIP1/RIP3), indicating that this form of cell death was not necroptosis. MPP+-induced cell death was also inhibited by several inhibitors of ferroptosis, including ferrostatin-1 (Fer-1). Although MPP+-induced death and ferroptosis shared some features, such as occurrence of lipid peroxidation and inhibition by Fer-1, MPP+-induced death seemed to be distinct from ferroptosis because MPP+-induced death (but not ferroptosis) was inhibited by Nec-1, was independent of p53, and was accompanied by ATP depletion and mitochondrial swelling. Further investigation of MPP+-induced non-apoptotic cell death may be useful for understanding the mechanisms of neuronal loss and for treatment of neurodegenerative diseases such as Parkinson’s disease.

## Introduction

Cell death has a critical role in various diseases, including neurodegenerative diseases, and is therefore an important therapeutic target, but little is known about the mechanisms of cell death associated with neurodegenerative diseases.^1–4^ It is now widely recognized that apoptosis is not the only form of regulated cell death, as there are also regulated types of necrotic death including necroptosis, ferroptosis, and autophagic death.^5^ Necroptosis is a death receptor-triggered form of necrotic cell death, which is mediated by activation of receptor-interacting serine/threonine-protein kinase 1/3 (RIP1 and RIP3), leading to oligomerization of mixed lineage kinase domain-like protein and its insertion into the plasma membrane.^6^ Necrostatin-1 (Nec-1) prevents necroptosis by binding to and inactivating RIP1.^7,8^ Ferroptosis is another genetically regulated form of necrotic cell death that is activated by several inducers, including erastin and RSL3, which promote iron-dependent lipid peroxidation by inhibiting system Xc- (cysteine/glutamate anti-transporter) and glutathione peroxidase 4, respectively.^9–11^ There are several known inhibitors of ferroptosis, such as the iron chelator deferoxamine (DFO) as well as ferrostatin-1 (Fer-1) and Trolox, which are scavengers of reactive oxygen species (ROS) to lipid. Oxidative stress is believed to be the principal cause of cell death due to ferroptosis, but the detailed mechanism remains unclear.

Parkinson’s disease (PD) is the second most common progressive neurodegenerative disease after Alzheimer’s disease. On pathological examination, patients with PD show loss of dopaminergic neurons in the pars compacta of the substantia nigra.^12,13^ Mitochondrial dysfunction is thought to be the main cause of neuronal death in PD, because many of the causative genes of familial PD discovered so far encode proteins involved in mitochondrial maintenance, such as PINK1 and Parkin.^14–16^ However, the mechanism leading to the death of dopaminergic neurons remains to be elucidated.

The compound 1-methyl-4-phenyl-1,2,3,6-tetrahydro-pyridine (MPTP) causes a disease state resembling PD in mammals, including humans.^17^ MPTP is converted to 1-methyl-4-phenylpyridinium (MPP+) by monoamine oxidase B in non-neuronal cells, such as glial cells and astrocytes, after which MPP+ causes selective impairment of dopaminergic neurons.^18,19^ It is thought that MPP+ affects mitochondrial complex I and causes ATP depletion like rotenone (a specific mitochondrial complex I inhibitor), and that it indirectly stimulates ROS production by triggering leakage of dopamine into the cytosol from synaptic vesicles, resulting in induction of apoptosis in dopaminergic neurons.^20–22^ P53 may also have a role in MPTP-induced neuronal apoptosis because death of dopaminergic neurons induced by MPTP is partially blocked by deletion of *p53*.^18,23,24^ However, it was recently reported that MPTP and MPP+ induce necrosis *in vivo* and *in vitro*, respectively.^25,26^ Therefore, the mechanism of cell death induced by MPTP/MPP+ is still controversial.

To obtain some insight into the mechanisms of cell death involved in neurodegenerative diseases, we analyzed MPP+-induced death of SH-SY5Y cells with neuronal differentiation. We found that MPP+-induced death was mainly p53-independent and non-apoptotic. It was inhibited by Nec-1, as well as by 3,3’-diindolylmethane (DIM) and some ferroptosis inhibitors such as Fer-1. Moreover, it was accompanied by changes of mitochondrial morphology and by lipid peroxidation. Although MPP+-induced death and ferroptosis shared some features, our findings suggest that these are two distinct mechanisms of cell death.

## Results

### MPP+ predominantly induces non-apoptotic cell death, which is strongly inhibited by Nec-1 and DIM

**Figure 1:**
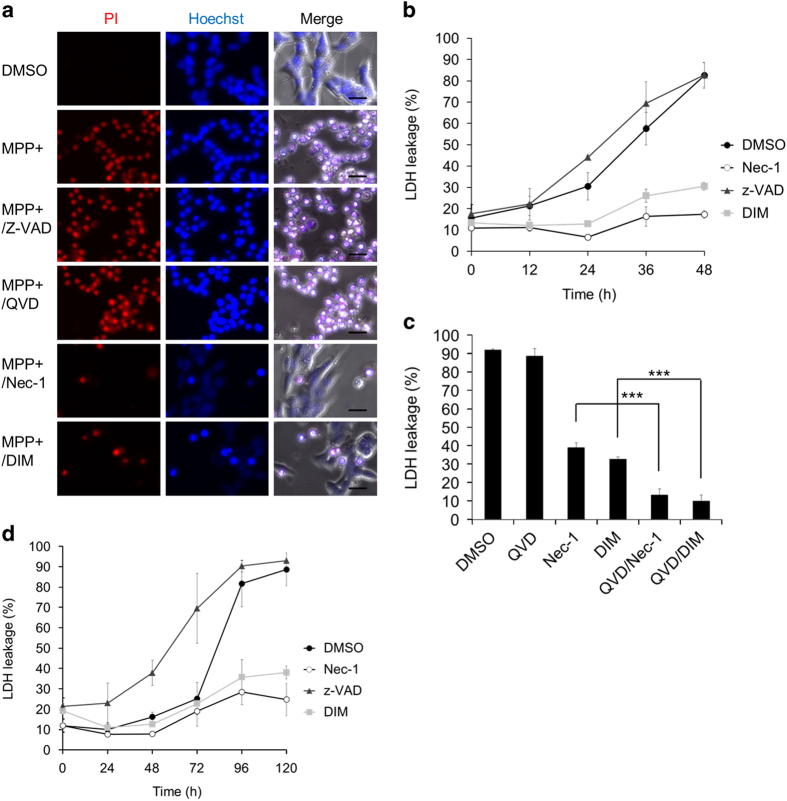
MPP+ induces non-apoptotic cell death, which is inhibited by Nec-1 and DIM. Neuronal SH-SY5Y cells were pretreated with inhibitors 30 min before the addition of MPP+ or rotenone. The inhibitors were Z-VAD-FMK (Z-VAD, 50 *μ*M), QVD-OPh (QVD, 10 *μ*M), Nec-1 (20 *μ*M), and DIM (20 *μ*M). (**a**) Cells were treated with MPP+ (5 mM) for 48 h and stained by adding PI (1 *μ*g/ml)/Hoechst 33342 (1 *μ*g/ml) to the culture medium. Scale bar=20 *μ*m. (**b** and **c**) Cells were treated with MPP+ (5 mM) for the indicated time (**b**) or 48 h (**c**) with inhibitors or dimethyl sulfoxide (DMSO) (control). Cell death was calculated from lactate dehydrogenase (LDH) leakage. (**d**) Cells were treated with rotenone (100 nM) for the indicated time. Cell death was calculated from LDH leakage. Data are shown as the mean±S.D. of three independent experiments. ***P<0.001.

SH-SY5Y cells were incubated with retinoic acid (RA) and then incubated with brain-derived neurotrophic factor (BDNF) to promote neuronal differentiation, as described in the Materials and Methods section. Proliferation of RA/BDNF-treated SH-SY5Y cells was almost completely suppressed when assessed by EdU incorporation, indicating successful neuronal differentiation (Supplementary Figures 1A and B).^27–29^ The differentiated SH-SY5Y cells were designated as neuronal SH-SY5Y cells. MPP+ induced the death of neuronal SH-SY5Y cells, as shown by double staining with PI/Hoechst 33342 (PI/Hoechst) (Figure 1a) and by the LDH release assay (Figure 1b). This form of cell death was not inhibited by pancaspase inhibitors, Z-VAD-FMK (Z-VAD) and QVD-OPh (QVD), indicating that MPP+ induced non-apoptotic death. By screening various agents, we found that Nec-1 and DIM strongly inhibited MPP+-induced death (Figures 1a–c). Nec-1 is a specific inhibitor of necroptosis that acts by directly blocking RIP1, and was also recently reported to inhibit certain other forms of cell death via an unknown mechanism.^30–32^ DIM is a compound derived from indole-3-carbinol, which is found in cruciferous vegetables and has antitumor activity.^33,34^ Although longer treatment was necessary, lower doses of MPP+ (such as 100 *μ*M) induced the death of neuronal SH-SY5Y cells, which was inhibited by Nec-1 and DIM, but not Z-VAD (Supplementary Figure 1C). Rotenone, a membrane-permeable inhibitor of mitochondrial complex I, also induced cell death that was inhibited by Nec-1 and DIM, but not by Z-VAD (Figure 1d). In addition, MPP+-induced cell death was almost completely inhibited by cotreatment with Nec-1/DIM and QVD (Figure 1c), indicating that inhibition of the death of neuronal SH-SY5Y cells by Nec-1/DIM activated apoptosis in a restricted small population of the cells. These findings suggested that MPP+ and rotenone, both of which are mitochondrial complex I inhibitors, predominantly induced non-apoptotic death of neuronal SH-SY5Y cells, which was inhibited by Nec-1 and DIM.

### MPP+-induced cell death is not inhibited by silencing RIP1 and RIP3

**Figure 2:**
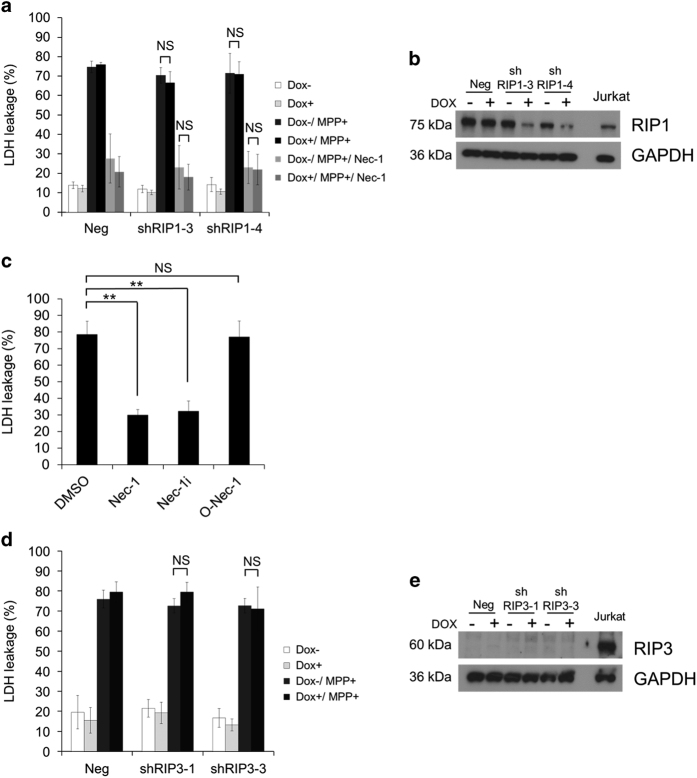
MPP+-induced cell death is not dependent on necroptosis. (**a** and **b**) After treatment of SH-SY5Y cells with RA, RIP1 short hairpin RNA (shRNA) was induced by incubation with Dox (2 *μ*g/ml) for 5 days in the presence of BDNF. (**a**) Dox-treated neuronal cells were incubated with MPP+ (5 mM) for 48 h in the presence or absence of Nec-1 (20 *μ*M). Cell death was calculated from lactate dehydrogenase (LDH) leakage. (**b**) RIP1 and glyceraldehyde 3-phosphate dehydrogenase (GAPDH) were detected by western blotting. The extreme right lane is Jurkat cell lysate (positive control). (**c**) Cells were treated with MPP+ in the presence of Nec-1 or its derivatives (20 *μ*M each) for 48 h. Cell death was calculated from LDH leakage. (**d** and **e**) After treatment of SH-SY5Y cells with RA, RIP3 shRNA was induced by incubation with Dox (2 *μ*g/ml) for 5 days in the presence of BDNF. (**d**) Dox-treated neuronal cells were incubated with MPP+ (5 mM) for 48 h. Cell death was calculated from LDH leakage. (**e**) RIP3 and GAPDH were detected by western blotting. The extreme right lane is Jurkat cell lysate (positive control). Data are shown as the mean±S.D. of three independent experiments. NS, not significant; **P<0.01.

Because MPP+-induced cell death was prevented by Nec-1, this raised the possibility that it might represent necroptosis mediated by RIP1 and RIP3. To investigate this point, we silenced RIP1 or RIP3 with tet-on inducible shRNA. Reduced expression of RIP1 due to RIP1 shRNA did not affect either MPP+-induced cell death or the inhibitory effect of Nec-1 (Figures 2a and b), suggesting that MPP+-induced death was not necroptosis and involved Nec-1-targeting molecule(s) other than RIP1. Supporting this result, MPP+-induced cell death was inhibited by an inactive derivative of Nec-1 (Nec-1i), which has no effect on necroptosis, but was not prevented by an active derivative (O-Nec-1) that inhibits necroptosis (Figure 2c).^35^

Expression of RIP3 was not detected in neuronal SH-SY5Y cells or in proliferating SH-SY5Y cells, as reported previously.^36^ Therefore, MPP+-induced cell death was unaffected by silencing RIP3, as expected (Figures 2d and e). RIP3 shRNA was validated in Jurkat cells, a human T-lymphocyte cell line (Supplementary Figure 2). These results demonstrated that necroptosis has no role in MPP+-induced non-apoptotic cell death and that Nec-1 prevents MPP+-induced death independently of RIP1.

### MPP+ induces mitochondrial changes that are not inhibited by Nec-1

**Figure 3:**
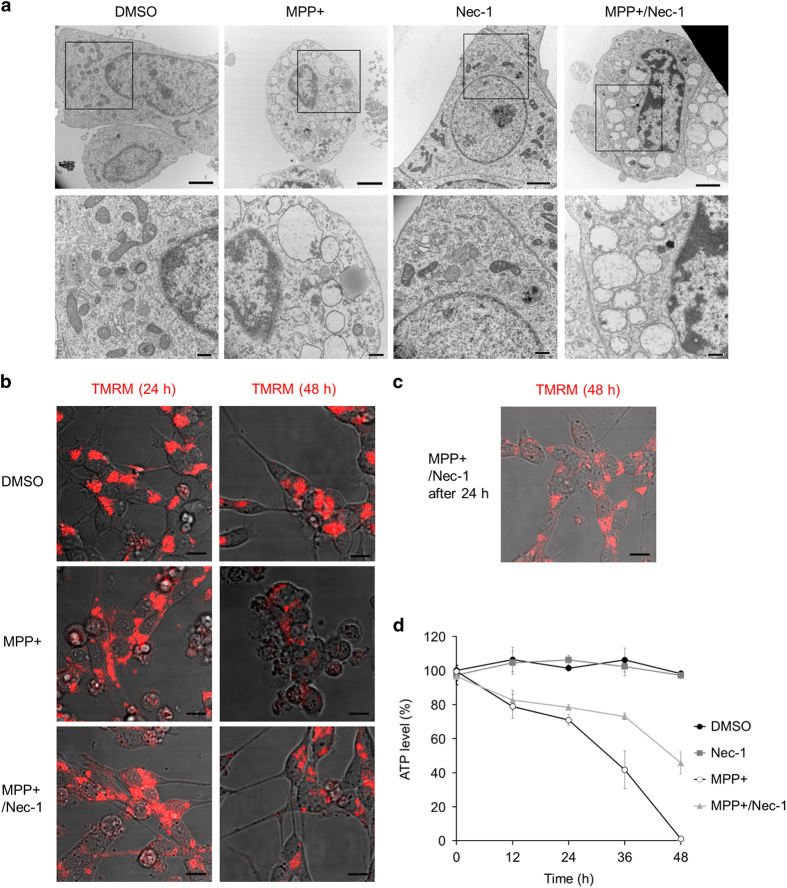
Changes of morphology and mitochondrial membrane potential during MPP+-induced cell death. (**a**) Neuronal SH-SY5Y cells were treated with MPP+ (5 mM) or dimethyl sulfoxide (DMSO) (control) for 40 h in the presence or absence of Nec-1 (20 *μ*M) and subjected to transmission electron microscopy. Scale bar=2 *μ*m (upper photos) or 500 nm (lower photos). (**b**) Representative TMRM staining to assess the mitochondrial membrane potential in neuronal SH-SY5Y cells. Cells were treated with MPP+ (5 mM) or DMSO (control) in the presence or absence of Nec-1 (20 *μ*M) for 24 h (left) or 48 h (right), stained with TMRM (250 nM), and subjected to confocal fluorescence microscopy. Scale bar=10 *μ*m. (**c**) Cells were treated with MPP+ (5 mM) for 48 h and Nec-1 (20 *μ*M) was added after 24 h treatment of MPP+. Scale bar=10 *μ*m. (**d**) Cells were treated with or without MPP+ (5 mM) in the presence or absence of Nec-1 (20 *μ*M). ATP was measured by the CellTiter-Glo cell viability assay at the indicated time. Data are shown as the mean±S.D. of three independent experiments.

We examined morphological changes after treatment of cells with MPP+ by transmission electron microscopy. In agreement with the results shown in Figure 1, few apoptotic cells were found. After MPP+ treatment, almost all of the neuronal cells contained numerous cytoplasmic vacuoles and a few normal mitochondria. In addition, the electron density of the cytosol was decreased, but there was no condensation of chromatin (Figure 3a). These findings are typical features of necrosis.^37^ Unexpectedly, there were also only a few normal mitochondria even after Nec-1 or DIM treatment (Figure 3a and Supplementary Figure 3A), suggesting that Nec-1 and DIM inhibited MPP+-induced cell death despite mitochondrial changes. To examine the effects of Nec-1 and DIM on the mitochondria in more detail, we monitored the mitochondrial membrane potential by TMRM staining. While the membrane potential decreased after 48 h of incubation without Nec-1 or DIM, it was relatively well maintained in the presence of either agent, although the TMRM signal was lower than in control cells (Figure 3b and Supplementary Figure 3B). We also found that addition of Nec-1-containing culture medium to neuronal cells after 24 h of incubation with MPP+ inhibited the MPP+-induced loss of the mitochondrial membrane potential (Figure 3c).

MPP+ is known to inhibit ATP production in the mitochondria by a direct effect on mitochondrial complex I.^19–22^ Therefore, we next analyzed the effect of Nec-1 on intracellular ATP levels by the CellTiter-Glo cell viability assay, revealing that intracellular ATP decreased over time during MPP+ treatment and that this change was significantly (but not completely) inhibited by Nec-1 (Figure 3d). These results indicated that Nec-1 and DIM both contribute to maintenance of the mitochondrial membrane potential and intracellular energy levels, but do not prevent morphological changes of the mitochondria induced by MPP+ treatment.

### MPP+-induced non-apoptotic cell death is independent of p53

**Figure 4:**
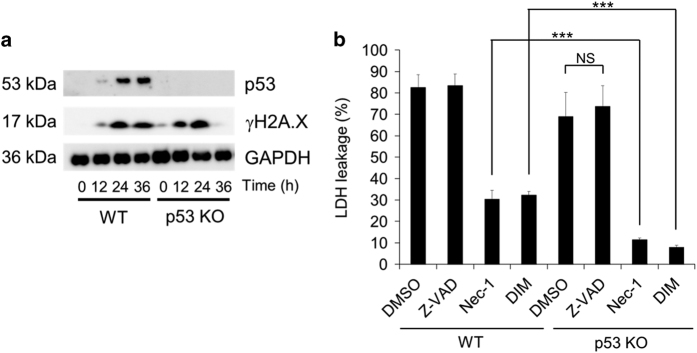
MPP+ induces p53-independent cell death, which is blocked by Nec-1 and DIM. (**a**) Lysates were obtained from WT and p53-deficient neuronal SH-SY5Y cells, which were treated with MPP+ (5 mM) for the indicated time. Then, p53, *γ*H2A.X, and GAPDH were detected by western blotting. (**b**) WT and p53-deficient neuronal SH-SY5Y cells were treated with MPP+ (5 mM) in the presence of Z-VAD (50 *μ*M), Nec-1 (20 *μ*M), DIM (20 *μ*M), or dimethyl sulfoxide (DMSO) (control) for 48 h. Cell death was calculated from lactate dehydrogenase (LDH) leakage. Data are shown as the mean±S.D. of three independent experiments. NS, not significant; ***P<0.001.

It is known that p53 not only has a role in apoptosis but is also involved in non-apoptotic cell death in various disease models,^38,39^ and p53 was reported to be crucial for MPP+/MPTP-induced neuronal loss.^18,23,24^ To investigate the possible contribution of p53 to MPP+-induced necrotic death of neuronal SH-SY5Y cells, we generated p53-deficient neuronal SH-SY5Y cells by using the CRISPR/Cas9 system (Supplementary Figure 4A). In addition to p53 protein not being detected by western blotting (Figure 4a), we attempted to confirm that p53 function was abolished in these p53-deficient SH-SY5Y cells by using etoposide to induce p53-dependent apoptosis. Etoposide triggered accumulation of p53 and led to Z-VAD-sensitive cell death in proliferating wild-type (WT) SH-SY5Y cells, but not in proliferating p53-deficient SH-SY5Y cells (Supplementary Figure 4B), confirming that there was no p53 activity to induce proapoptotic proteins such as Puma in the latter cells. MPP+ caused accumulation of p53 in WT cells, but not in p53-deficient cells, while accumulation of *γ*H2A.X (a marker of DNA damage) occurred in both cell types (Figure 4a). MPP+-induced cell death was only slightly reduced in p53-deficient cells (Figure 4b), although death of these cells was almost completely inhibited by Nec-1 or DIM (Figure 4b). These findings, taken together with the results shown in Figure 1c (almost complete inhibition of MPP+-induced death of WT neuronal SH-SY5Y cells by Nec-1/DIM plus QVD), suggest that MPP+ induced two forms of death in neuronal SH-SY5Y cells, with p53-dependent death occurring in a small-cell population and most cells undergoing p53-independent non-apoptotic death that was inhibited by Nec-1 and DIM.

### MPP+-induced non-apoptotic death is blocked by ferroptosis inhibitors

**Figure 5:**
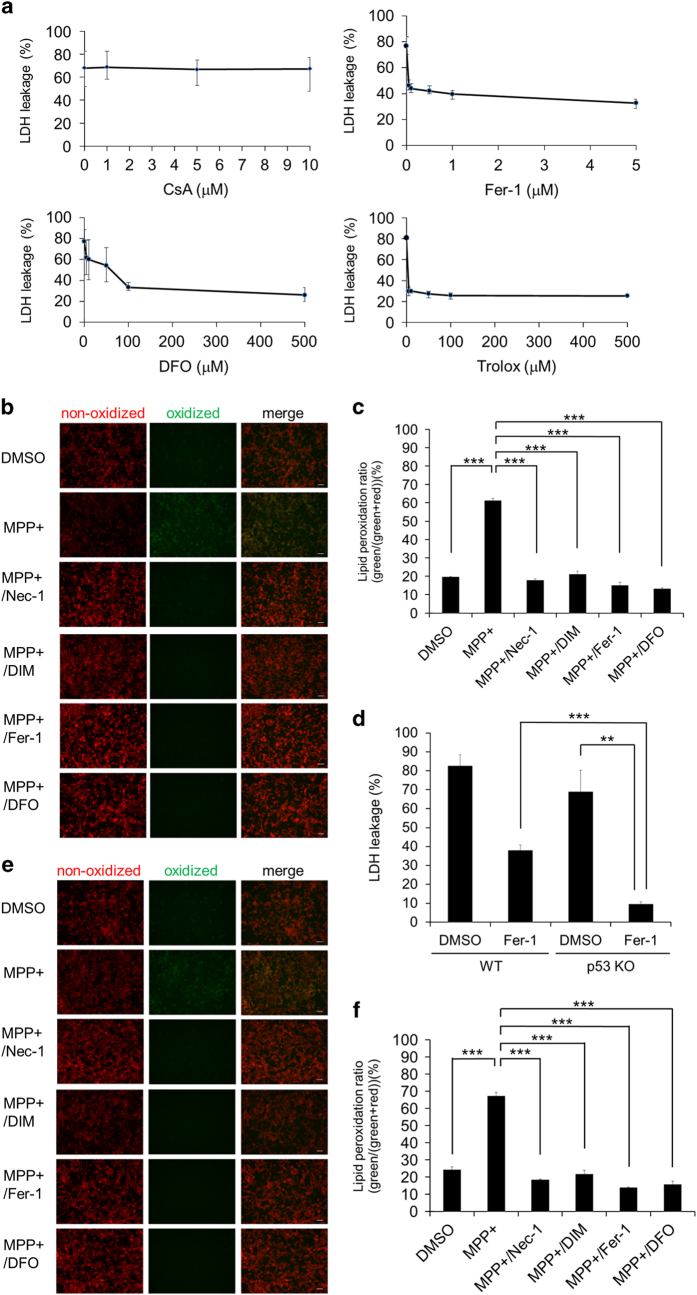
MPP+-induced cell death is inhibited by ferroptosis inhibitors, but not by CsA. (**a**) Neuronal SH-SY5Y cells were treated for 48 h with MPP+ (5 mM) in the presence of a CypD-dependent cell death inhibitor (CsA, upper left), ferroptosis inhibitors (Fer-1, upper right; DFO, lower left; Trolox, lower right) at the indicated concentrations. Cell death was calculated from lactate dehydrogenase (LDH) leakage. (**b**) Representative C11-BODIPY staining of lipids in the membrane of WT neuronal SH-SY5Y cells. Red signals indicate non-oxidized lipids and green signals indicate oxidized lipids. Cells were treated with MPP+ (5 mM) in the presence of Nec-1 (20 *μ*M), DIM (20 *μ*M), Fer-1 (2 *μ*M), DFO (100 *μ*M), or dimethyl sulfoxide (DMSO) (control) for 48 h, and subjected to fluorescence microscopy. Scale bar=50 *μ*m. (**c**) Red and green signals in (**b**) were quantified by using the Image J software. The lipid peroxidation ratio was calculated as follows: mean value for green signals/(mean value for red signals+mean value for green signals). Each cell field was selected by the minimum error algorithm. (**d**) WT and p53-deficient neuronal SH-SY5Y cells were treated with MPP+ in the presence or absence of Fer-1 (2 *μ*M) for 48 h. Cell death was calculated from LDH leakage. (**e**) Representative C11-BODIPY staining of lipids in membrane of p53-deficient neuronal SH-SY5Y cells. Red signals and green signals indicate non-oxidized and oxidized lipids, respectively. Cells were treated with MPP+ (5 mM) in the presence of Nec-1 (20 *μ*M), DIM (20 *μ*M), Fer-1 (2 *μ*M), DFO (100 *μ*M), or DMSO (control) for 48 h, and subjected to fluorescence microscopy. Scale bar=50 *μ*m. (**f**) Red and green signals in (**e**) were quantified by using the Image J software. The lipid peroxidation ratio was calculated as follows: mean value for green signals/(mean value for red signals+mean value for green signals). Each cell field was selected by the minimum error algorithm. Data are shown as the mean±S.D. of three independent experiments. **P<0.01; ***P<0.001.

Several forms of non-apoptotic (necrotic) cell death have been reported, including ferroptosis ^9–11^ and cyclophilin D (CypD)-dependent mitochondrial permeability transition-triggered necrosis.^40^ We investigated whether MPP+-induced death was related to any of these forms of necrotic cell death. Because MPP+-induced death was accompanied by mitochondrial membrane potential loss, we first tested the possible involvement of CypD-dependent necrosis, which is known to be accompanied by mitochondrial membrane potential loss. For this purpose, we used cyclosporin A (CsA), a specific inhibitor of this mode of necrosis that forms a complex with CypD and inhibits its activity.^40,41^ CsA did not inhibit MPP+-induced death of neuronal SH-SY5Y cells (Figure 5a), excluding the involvement of CypD-dependent necrosis in MPP+-induced death. We next examined ferroptosis, because peroxidation of lipids has a crucial role in ferroptosis^9–11^ and MPP+ has a potential to produce oxygen radicals.^42^ We found that MPP+-induced cell death was strongly inhibited by several ferroptosis inhibitors, including Fer-1, DFO, and Trolox (Figure 5a). Fer-1 is an aromatic amine that specifically binds with lipid ROS and protects cells from lipid peroxidation. DFO has a high affinity for extracellular free iron, which is directly involved in ROS production. Trolox is a hydrophilic analog of vitamin E with antioxidant activity like the vitamin E itself and it protects cells from damage due to oxidative stress.^9–11^ The results suggested some relationship of MPP+-induced death to ferroptosis, especially involvement of lipid ROS.

We then directly investigated lipid peroxidation during MPP+-induced death, as lipid peroxidation is considered to be the main cause of ferroptosis. By staining unoxidized/oxidized lipid with C11-BODIPY, we found that MPP+-induced death was accompanied by accumulation of oxidized lipid, while lipid accumulation was strongly inhibited by ferroptosis inhibitors as well as by Nec-1 and DIM (Figures 5b and c). Thus, MPP+-induced cell death and ferroptosis shared some features as both were inhibited by Fer-1, DFO, and Trolox, and both were associated with lipid peroxidation. Despite these similarities, there were also several differences between MPP+-induced death and ferroptosis. First, MPP+-induced cell death, but not ferroptosis, was inhibited by Nec-1, while we confirmed that Nec-1 did not inhibit either ferroptosis (death) or accompanying lipid peroxidation induced by erastin or RSL3 in HT1080 cells (a human fibrosarcoma cell line) and mouse embryonic fibroblasts (MEFs) (Supplementary Figures 5A–D). Other differences between the two forms of cell death were that MPP+-induced death was associated with mitochondrial swelling, ATP depletion, and p53-independence, none of which were observed with ferroptosis.^9^ It was reported that p53 sensitizes cells to ferroptosis by downregulating expression of *SLC7A11*, which encodes a component of system Xc−, at the transcriptional level,^43^ activating SAT1 through ALOX15,^44^ and activating glutaminase 2.^45^ On the other hand, MPP+-induced death of p53-deficient cells was also prevented by Fer-1 (Figure 5d), like Nec-1 and DIM (Figure 4). In agreement with inhibitors of MPP+-induced death, lipid peroxidation was prevented by Fer-1 and DFO in p53-deficient cells (Figures 5e and f). These results suggested that the two forms of cell death differed with respect to p53 dependency.

**Figure 6:**
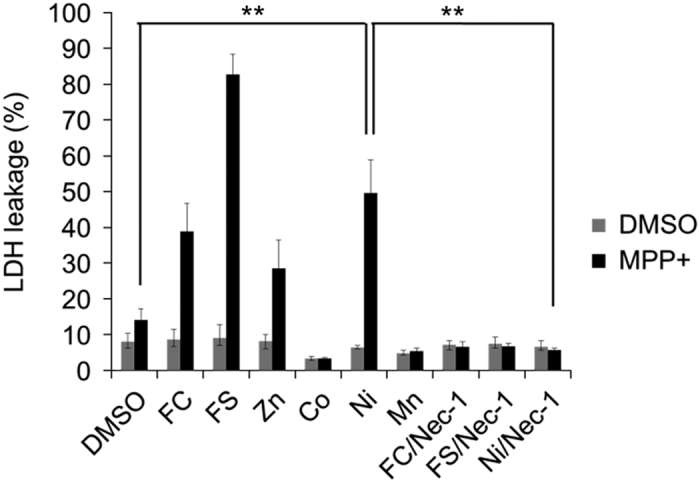
MPP+-induced cell death is enhanced by metal ions. Viability of p53-deficient neuronal SH-SY5Y cells incubated for 48 h with or without MPP+ (0.1 mM) in the presence of Nec-1 (20 *μ*M) or various metal ions (25 *μ*M), including Fe^3+^ derived from FC, Fe^2+^ from ferrous sulfate (FS), Zn^2+^ from zinc nitrate hexahydrate (Zn), Co^2+^ from cobalt chloride hexahydrate (Co), Ni^2+^ from nickel sulfate hexahydrate (Ni) and Mn^2+^ from manganese chloride (Mn). Cell death was calculated from LDH leakage. Data are shown as the mean±S.D. of three independent experiments. **P<0.01.

We further investigated the relationship between MPP+-induced cell death and ferroptosis as follows. It was previously reported that increasing the concentration of trivalent ionized iron, but not other divalent metal ions, accelerated erastin-induced ferroptosis.^9^ Therefore, we examined the effects of various metal ions on these two forms of cell death. All of the ions tested did not show any toxicity for neuronal SH-SY5Y cells (Figure 6) or MEFs (Supplementary Figure 6). To examine whether MPP+-induced cell death was enhanced by metal ions, we used 0.1 mM MPP+, which induced the same Nec-1/DIM-sensitive necrotic cell death as 5 mM MPP+ although lower extent (Supplementary Figure 1C). Both erastin-induced ferroptosis of MEFs and MPP+-induced death of p53-deficient neuronal SH-SY5Y cells were reinforced by Fe^3+^ (derived from ferric citrate (FC)), Fe^2+^ obtained from ferrous sulfate heptahydrate (FS) and Zn^2+^ from zinc nitrate hexahydrate (Zn), but were strongly inhibited by Co^2+^ from cobalt chloride hexahydrate (Co) and Mn^2+^ from manganese chloride (Mn) (Figure 6 and Supplementary Figure 6), whereas MPP+-induced death, but not ferroptosis, was reinforced by Ni^2+^ from nickel sulfate hexahydrate (Ni) (Figure 6 and Supplementary Figure 6). Nec-1 prevented Fe^3+^-, Fe^2+^-, and Ni^2+^-accelerated MPP+-induced death (Figure 6). Therefore, both MPP+-induced necrotic death of neuronal SH-SY5Y cells and erastin-induced ferroptosis of MEFs were accelerated by iron ions and Zn^2+^, but only MPP+-induced necrotic death was promoted by Ni^2+^.

*N*-acetyl-l-cysteine (NAC) is a well-known ROS scavenger and it prevents FBS-dependent ferroptosis in MEFs.^46^ Consistent with previous reports, NAC prevented lipid peroxidation and death after treatment of MEFs and HT1080 cells with erastin or RSL3 (Supplementary Figures 5A–D). However, MPP+-induced death and lipid peroxidation of neuronal SH-SY5Y cells was not inhibited by NAC (Supplementary Figures 7A and B).

**Figure 7:**
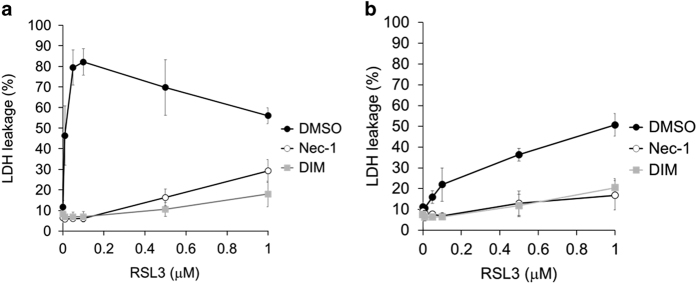
Analysis of RSL3-induced death of neuronal SH-SY5Y cells. (**a**) WT neuronal SH-SY5Y cells and (**b**) p53-deficient neuronal SH-SY5Y cells were incubated for 72 h with RSL3 at the indicated concentrations in the presence of Nec-1 (20 *μ*M), DIM (20 *μ*M), or dimethyl sulfoxide (DMSO) (control). Cell death was calculated from LDH leakage. Data are shown as the mean±S.D. of three independent experiments.

Finally, we examined whether ferroptosis inducers could cause the death of neuronal SH-SY5Y cells. We found that RSL3, but not erastin, induced the death of neuronal SH-SY5Y cells, which was prevented by Nec-1 and DIM (Figure 7a). RSL3 induced the death of WT neuronal SH-SY5Y cells at a lower concentration than the level that was effective against p53-deficient neuronal cells, indicating p53-sensitivity of RSL3-induced death (Figures 7a and b). Nec-1 and DIM also inhibited RSL3-induced death in p53-deficient neuronal SH-SY5Y cells (Figure 7b).

Altogether, MPP+-induced cell death and ferroptosis shared some features (both were inhibited by several agents including Fer-1 and DIM, both were accompanied by lipid peroxidation, and both showed sensitivity to iron ions and Zn^2+^), there were also significant differences with regard to p53 dependence, NAC sensitivity, inhibition by Nec-1, mitochondrial morphology, changes of ATP levels, and Ni^2+^ sensitivity, suggesting that MPP+-induced death of neuronal SH-SY5Y cells had a distinct mechanism from ferroptosis.

## Discussion

To obtain some insight into the mechanism of neuronal death leading to neurodegenerative diseases, we used neuronally differentiated SH-SY5Y cells in the present study. We found that MPP+ induced non-apoptotic death of these cells that was inhibited by Nec-1 and DIM, although it also induced p53-dependent apoptosis in a small-cell population (Figures 1 and 4).

Mitochondria provide energy to maintain neuronal activity, and several genes associated with familial PD, such as *parkin* and *pink1*, are related to mitochondrial maintenance systems.^47^ MPP+ and rotenone cause mitochondrial dysfunction and are thought to inhibit ATP synthesis and promote ROS generation, eventually resulting in cell death. We demonstrated that MPP+-induced cell death was accompanied by mitochondrial swelling, which was not inhibited by Nec-1 or DIM, suggesting that both of these agents acted downstream of MPP+-induced mitochondrial morphological changes. We also showed that the mitochondrial membrane potential was reduced by MPP+ treatment, while this decrease was significantly inhibited by Nec-1 and DIM. Since most mitochondria still exhibited swelling in the presence of Nec-1 and DIM, preservation of the membrane potential was probably due to the reverse action of FoF_1_ ATPase using cytoplasmic ATP, and suggested that the cells were still viable. We further demonstrated that MPP+-induced cell death was accompanied by lipid peroxidation, which was also inhibited by Nec-1 and DIM. Despite some previous observations suggesting that p53 might have a role in MPP+-induced death,^23,24^ we found that MPP+-induced non-apoptotic cell death was independent of p53.

Several forms of necrotic programmed cell death have been described, so we investigated whether MPP+-induced death corresponded to any of the known types of necrotic death. We showed that MPP+-induced death was distinct from necroptosis, because it was independent of RIP1/RIP3, and distinct from CypD-dependent necrosis, because it was not inhibited by CsA.

Although ferroptosis is unaffected by Nec-1, which strongly inhibited MPP+-induced death, we found that MPP+-induced death was also strongly inhibited by several ferroptosis inhibitors (Fer-1, DFO, and Trolox), raising the possibility that MPP+-induced death corresponded to ferroptosis. In two cell lines (HT1080 and MEF) that are widely used to study ferroptosis, we confirmed that erastin-induced ferroptosis was not inhibited by Nec-1, but was strongly inhibited by Fer-1 and was also inhibited by DIM. Furthermore, we found that exogenous free iron ions or Zn^2+^ enhanced both MPP+-induced death and ferroptosis, and that MPP+-induced death and ferroptosis were strongly inhibited by both Co^2+^ and Mn^2+^.

Thus, MPP+-induced cell death and ferroptosis shared some features, including inhibition by DIM and Fer-1, occurrence of lipid peroxidation, enhancement by iron ions or Zn^2+^, and inhibition by Co^2+^ and Mn^2+^. However, these two forms of cell death also exhibited the following differences: (1) Nec-1 only inhibited MPP+-induced death, (2) NAC only inhibited ferroptosis, (3) different changes of mitochondrial morphology (swelling in MPP+-induced death *versus* being small mitochondria in ferroptosis),^9^ (4) differences of ATP (depletion in MPP+-induced death *versus* no change in ferroptosis),^9^ and (5) only MPP+-induced death was sensitized by Ni^2+^. These findings strongly suggest that MPP+-induced death is different from ferroptosis. We also revealed that RSL3, but not erastin, induced the death of neuronal SH-SY5Y cells, which was inhibited by DIM and by Nec-1. These findings might imply that RSL3 induces ferroptosis of neuronal SH-SY5Y cells, while inhibition by Nec-1 is dependent on the cellular context. Alternatively, RSL3 might activate a similar type of cell death as that induced by MPP+ in neuronal SH-SY5Y cells rather than ferroptosis.

It has been reported that accumulation of iron is common at sites of central nervous system pathology,^48,49^ and mice with MPTP-induced Parkinsonism were rescued by treatment with Fer-1 and an iron chelator deferiprone,^50,51^ suggesting that iron-associated necrotic cell death and lipid peroxidation may be associated with the loss of dopaminergic neurons in PD. Although it has been reported that ferroptosis is involved in neuronal death in the animal model of MPTP-induced Parkinsonism, it might be that the cell death actually corresponds to MPP+-induced death described here.

In summary, MPP+-induced necrotic cell death and ferroptosis shared some features, and also exhibited many differences, including p53 dependence, NAC sensitivity, inhibition by Nec-1, mitochondrial morphology, changes of ATP, and Ni^2+^ sensitivity, suggesting that MPP+-induced necrotic death of neuronal SH-SY5Y cells is a distinct process from ferroptosis. Further studies are needed to identify genes specifically involved in MPP+-induced death and/or ferroptosis, which could help to better understand the pathogenesis of PD.

## Materials and Methods

### Chemicals

Nec-1, an Nec-1i, and O-necrostatin-1 (5-(1*H*-indol-3-ylmethyl)hydantoin necrostatin, O-Nec-1) were synthesized. The pancaspase inhibitors Z-VAD-FMK and QVD-OPh were purchased from Peptide Institute Inc. (Ibaraki, Osaka, Japan) and Millipore (Darmstadt, Germany), respectively. Erastin and RSL3 were obtained from Sigma-Aldrich (St Louis, MO, USA) and Namiki Corporation (Shinjuku, Tokyo, Japan), respectively. All other chemicals were purchased from Sigma-Aldrich, unless otherwise stated.

### Cell lines and culture

SH-SY5Y cells were obtained from Dr. Masato Miyake (National Institute of Advanced Industrial Science and Technology), HT1080 cells were obtained from Dr. Kazuyuki Ito (Research Institute, Nozaki Tokusyukai Hospital), and MEFs were obtained from Dr. Shigeomi Shimizu (Tokyo Medical and Dental University). Proliferating SH-SY5Y cells were cultured in DMEM medium (Nacalai Tesque Inc., Kyoto, Japan) containing 10% FBS (Thermo Fisher Scientific K.K., Yokohama, Japan). For differentiation into neuronal cells, proliferating SH-SY5Y cells were cultured for 7 days with 10 *μ*M RA in DMEM/Ham’s F-12 medium containing 10% FBS, 2 mM glutamine, and antibiotics (penicillin and streptomycin), followed by culture for a further 5–6 days with 50 ng/ml BDNF (Aviscera Bioscience Inc., Santa Clara, CA, USA) in serum-free DMEM/Ham’s F-12 medium containing 2 mM glutamine and antibiotics. Neuronal cells were seeded in collagen-coated 24-well plates (LDH assay, nuclear staining, and detection of lipid peroxidation), 35 mm dishes (mitochondrial membrane potential), or 6-well plates (immunoblotting) at a density of 400 000 cells/cm^2^ in 500 *μ*l, 1 ml, or 2 ml of the appropriate serum-free media, respectively. HT1080 cells and MEFs were cultured in DMEM medium containing 10% FBS and were seeded in 24-well plates (LDH assay and detection of lipid peroxidation) at a density of 20 000 cells/cm^2^ in 500 *μ*l of medium.

### Cell death assay

Cell death was assessed with PI/Hoechst double nuclear staining: cells were incubated with 1 *μ*g/ml PI and 1 *μ*g/ml Hoechst 33342 for 30 min at 37 °C, and then were observed under a BZ-X710 all-in-one fluorescence microscope (Keyence Co., Osaka, Japan). Cell death was also determined by using the LDH Cytotoxicity Assay Kit (Wako Pure Chemical Industries, Ltd., Osaka, Japan) to measure LDH released from necrotic cells by rupture of the plasma membrane. Briefly, culture supernatants and cells were harvested separately. Culture supernatants were centrifuged for 2 min at 5000 r.p.m. and the supernatants were saved to new centrifuge tubes, whereas the precipitates including cells and debris were mixed with the harvested cells at room temperature and lysed by treatment with PBS/1 mM EDTA containing 0.1% Tween-20. Diluted supernatants and cell lysates were dispensed into 96-well plates, LDH reagents were added and LDH activity was measured using a microplate reader. Cell death was determined by dividing the LDH activity in the supernatant by that for the supernatant plus cell lysate.

### Immunoblotting

Cells were harvested and suspended in FLICE buffer, which contained 25 mM HEPES (pH 7.4), 1 mM EDTA, 0.1% CHAPS, 10% sucrose, protease inhibitor cocktail and phosphatase inhibitor cocktail (Roche Diagnostics K.K., Tokyo, Japan). Cells were lysed by adding 1% SDS. Then, the lysates were heated for 5 min at 98 °C and analyzed by SDS-PAGE, followed by western blotting using the following antibodies: anti-human RIP1 antibody (1 : 1000, monoclonal mouse IgG clone no. 334640; R&D Systems Inc., Minneapolis, MN, USA), anti-human RIP3 antibody (1 : 1000, monoclonal rabbit IgG no. 13526; Cell Signaling Technology Japan, K.K., Tokyo, Japan), anti-p53 antibody (7F5) (1 : 1000, monoclonal rabbit IgG no. 2527; Cell Signaling Technology Japan), anti-phosphor-histone H2A.X antibody (1 : 1000, monoclonal mouse IgG clone JBW301; Millipore), anti-GAPDH antibody (1 : 2000, monoclonal mouse IgG clone 6C5 (Millipore) or 1 : 2000, monoclonal rabbit IgG clone 14C10 (Cell Signaling Technology Japan)), HRP-conjugated anti-mouse antibody (1 : 2000, monoclonal goat IgG no. C2011; Santa Cruz Biotechnology, Dallas, TX, USA) and HRP-conjugated anti-rabbit antibody (1 : 2000, monoclonal goat IgG no. 7074; Cell Signaling Technology Japan).

### Detection of the mitochondrial membrane potential

The mitochondrial membrane potential was assessed by staining with TMRM (Cosmo Bio Co., Tokyo, Japan). Cells were incubated with TMRM (250 nM) for 30 min at 37 °C after culture with or without MPP+ for 24 or 48 h, and then were observed under a LSM510 ZEN confocal fluorescence microscope (Carl Zeiss Microscopy Co., Ltd., Tokyo, Japan).

### Measurement of ATP

The ATP level was determined by using the CellTiter-Glo Luminescent Assay Kit (Promega K.K., Tokyo, Japan) according to the manufacturer’s protocol.

### Transmission electron microscopy

RA-treated SH-SY5Y cells were seeded onto Thermanox Plastic Coverslips (Thermo Fisher Scientific), and were differentiated into neuronal cells with BDNF treatment. Cells were washed with D-PBS (−), fixed in 0.1 M sodium cacodylate buffer (pH 7.4) containing 2.5% glutaraldehyde overnight at 4 °C, and subsequently fixed in 0.1 M sodium cacodylate buffer (pH 7.4) containing 1% osmium tetroxide for 1 h at 4 °C. Fixed cells were stained *en bloc* with 0.5% uranium acetate. After embedding in Epon812 (TAAB), ultrathin sections (~90 nm) were cut, stained with aqueous lead citrate and uranyl acetate, and examined with an H7100 electron microscope (Hitachi, Tokyo, Japan) at an acceleration voltage of 75 kV.

### Silencing of RIP1 and RIP3

The tet-on system was used for expression of shRNA. HEK293T cells were co-transfected with a lentivirus expression vector (pRSI-U6T-(sh)-CMV-TetR-2A-mCherry-2A-Puro; Cellecta Inc., Mountain View, CA, USA), carrying shRNA sequences targeting RIP1 or RIP3 and the pC-Pack2 packaging plasmid (Cellecta Inc.). Proliferating SH-SY5Y cells were infected with the harvested virus carrying shRNA sequences. The target sequences are shown below. Transfected cells were selected by culture in medium containing 2 *μ*g/ml puromycin for 2 weeks. After treatment of SH-SY5Y cells with RA, RIP1/RIP3 shRNA was induced by incubation with 2 *μ*g/ml doxycycline (Dox) for 5 days in the presence of BDNF.

Target sequences were as follows: RIP1 (RIP1–3), 5′- TGCAGTCTCTTCAACTTGA-3′; RIP1 (RIP1–4), 5′-GAATGTGGCTTACAACAGA-3′; RIP3 (RIP3-1), 5′-CCAGAGACCTCAACTTTCA-3′; RIP3 (RIP3-3), 5′-GGGAGGTCAAGGCCATGGCAAGTCT-3′.

### Generation of p53-deficient cells with the CRISPR/Cas9 system

The CRISPR/Cas9 system was used to produce p53-deficient cells. The p53-knockout target sequence, which is shown below, was inserted into the pSpCas9(BB)-2A-Puro (pX459) vector (no. 48139; Addgene, Tokyo, Japan).^52^ Proliferating SH-SY5Y cells were transfected with this plasmid by electroporation with Neon (Thermo Fisher Scientific K.K.), and selected by culture in medium containing 2 *μ*g/ml puromycin for 48 h. Cells were replated in 96-well plates (0.5 cells per well) for cloning.

Target sequence was as follows: 5′-CGGACGATATTGAACAATGG-3′.

### Detection of lipid peroxidation

Cells were incubated in BDNF-containing culture medium with 5 *μ*M C11-BODIPY581/591 (Thermo Fisher Scientific) for 30 min at 37 °C, after which the medium was aspirated and BDNF-containing culture medium with or without each inhibitor was added. After another 30 min, medium containing various agents was added. Oxidation of C11-BODIPY581/591 was indicated by the change of BODIPY fluorescence from red (non-oxidized) to green (oxidized), and was observed under a BZ-X710 fluorescence microscope (Keyence Co.). Quantification of red and green signals was performed with the Image J software (US National Institutes of Health, Bethesda, MD, USA).

### Statistical analysis

Data are presented as the mean±S.D. from three independent experiments. Student’s *t*-test was used to determine the significance of differences between groups, and *P*<0.05 was considered to indicate statistical significance.
