# Supplementary material for: MPP+ induces necrostatin-1- and ferrostatin-1-sensitive necrotic death of neuronal SH-SY5Y cells
Source: Cell Death Discov. 2017 Feb 27;3:17013–. doi: 10.1038/cddiscovery.2017.13 (PMC5327502; doi:10.1038/cddiscovery.2017.13)

SUPPLEMENTARY INFORMATION

Legend of Supplementary Figures

Supplementary Figure 1 Differentiation of SH-SY5Y cells into neuronal cells (related to Figure 1).

(A) Representative photos of EdU uptake by untreated SH-SY5Y cells, retinoic acid (RA)-treated SH-SY5Y cells, and RA/BDNF-treated SH-SY5Y cells. Scale bar = 50 μm. (B) EdU uptake, indicating the proliferation rate, was measured in each group. Mean brightness was calculated by from the photographs in (A) using Image J software. (C) Cells were treated with MPP+ (0.1 mM) for the indicated time in the presence of Nec-1 (20 μM), Z-VAD (50 μM), DIM (20 μM), or DMSO (control). Cell death was calculated from LDH leakage. Data are shown as the mean +/- S.D. of three independent experiments. **P < 0.01; ***P < 0.001.

Supplementary Figure 2 Validation of shRNA for silencing RIP3 in Jurkat cells (related to Figure 2).

RIP3 shRNA was induced in Jurkat cells by incubation with Dox (1 μg/ml) for 5 days. RIP3 and GAPDH were detected by Western blotting.

Supplementary Figure 3 MPP+-induced loss of the mitochondrial membrane potential is

inhibited by DIM, but not morphological changes of the mitochondria (related to Figure 3).

(A) Neuronal SH-SY5Y cells were treated with MPP+ (5 mM) or DMSO (control) for 30 hours in the presence or absence of DIM (20 μM) and were subjected to transmission electron microscopy. Scale bar = 2 μm (upper photos) or 500 nm (lower photos). (B) Representative TMRM staining to show the mitochondrial membrane potential in neuronal SH-SY5Y cells. Cells were treated with MPP+ (5 mM) or DMSO (control) in the presence or absence of DIM (20 μM) for 48 hours, and subjected to confocal fluorescence microscopy. Scale bar = 10 μm.

Supplementary Figure 4 p53-deficient SH-SY5Y cells generated by the CRISPR/Cas9 system (related to Figure 4).

(A) Amino acid sequences surrounding the site of the mutation in p53 protein in WT and p53-deficient SH-SY5Y cells. (B) WT and p53-deficient proliferating SH-SY5Y cells were treated with etoposide (100 μM) in the presence of Nec-1 (20 μM), Z-VAD (50 μM), or DMSO (control) for 24 hours. Cell death was calculated from LDH leakage. Data are shown as the mean +/- S.D. of three independent experiments. ***$P < 0.001$.

Supplementary Figure 5 Inhibition of erastin- and RSL3-induced ferroptosis by DIM

and NAC, but not Nec-1 (related to Figure 5).

(A) HT1080 cells were treated with erastin (5 μM) in the presence of Fer-1 (2 μM), Nec-1 (20 μM), DIM (20 μM), NAC (5 mM), or DMSO (control) for 24 hours. (B) (left) Representative C11-BODIPY staining of lipids in membrane of HT1080 cells treated with erastin (5 μM) in the presence of Nec-1 (20 μM), DIM (20 μM), Fer-1 (2 μM), NAC (5 mM), or DMSO (control) for 8 hours. Red signals indicate non-oxidized membrane lipids and green signals indicate oxidized lipids (right). Red and green signals were quantified by using Image J software (left). The lipid peroxidation ratio was calculated as: mean value for green signals / (mean value for red signals + mean value for green signals). Cell fields were selected by the minimum error algorithm. (C) MEFs were treated with erastin (5 μM) or RSL3 (1 μM) for 24 hours. Cell death was calculated from LDH leakage. (D) (left) Representative C11-BODIPY staining of lipids in membrane of MEFs treated with erastin (5 μM) in the presence of Nec-1 (20 μM), DIM (20 μM), Fer-1 (2 μM), NAC (5 mM), or DMSO (control) for 8 hours. Red signals and green signals indicate non-oxidized or oxidized lipids, respectively (right). Red and green signals were quantified by using Image J software (left). The lipid peroxidation ratio was calculated as: mean value for green signals / (mean value for red signals + mean value for green signals). Cell fields were selected by the minimum error algorithm. Data are shown as the mean +/- S.D. of three independent experiments. NS: not significant; *$P < 0.05$; **$P < 0.01$; ***$P < 0.001$.

Supplementary Figure 6 Erastin-induced ferroptosis is enhanced by metal ions (related to Figure 6).

MEFs were incubated for 6 hours with or without erastin (5 μM) in the presence of Fer-1 (2 μM) or various metal ions (25 μM), including Fe^{3+} derived from ferric citrate (FC), Fe^{2+} from ferrous sulfate (FS), Zn^{2+} from zinc nitrate hexahydrate (Zn), Co^{2+} from cobalt chloride hexahydrate (Co), Ni^{2+} from nickel sulfate hexahydrate (Ni), and Mn^{2+} from manganese chloride (Mn). Cell death was calculated from LDH leakage. Data are shown as the mean +/- S.D. of three independent experiments. NS: not significant.

Supplementary Figure 7 MPP+-induced cell death and lipid peroxidation are not inhibited by NAC.

(A) WT neuronal SH-SY5Y cells were treated with MPP+ (5 mM) in the presence or absence of NAC (5 mM) for 48 hours. Cell death was calculated from LDH leakage. (B) (left) Representative C11-BODIPY staining of lipids in membrane of WT neuronal SH-SY5Y cells incubated with MPP+ (5 mM) for 48 hours in the presence or absence of NAC (5 mM). Red signals indicate non-oxidized membrane lipids and green signals indicate oxidized lipids (right). Scale bar = 50 μm. Red and green signals were quantified by using Image J software (left). The

lipid peroxidation ratio was calculated as: mean value for green signals / (mean value for red signals + mean value for green signals). Cell fields were selected by the minimum error algorithm. Data are shown as the mean +/- S.D. of three independent experiments. NS: not significant; **$P < 0.01$; ***$P < 0.001$.

Supplementary Figures

Supplementary Figure 1 Differentiation of SH-SY5Y cells into neuronal cells (related to Figure 1).

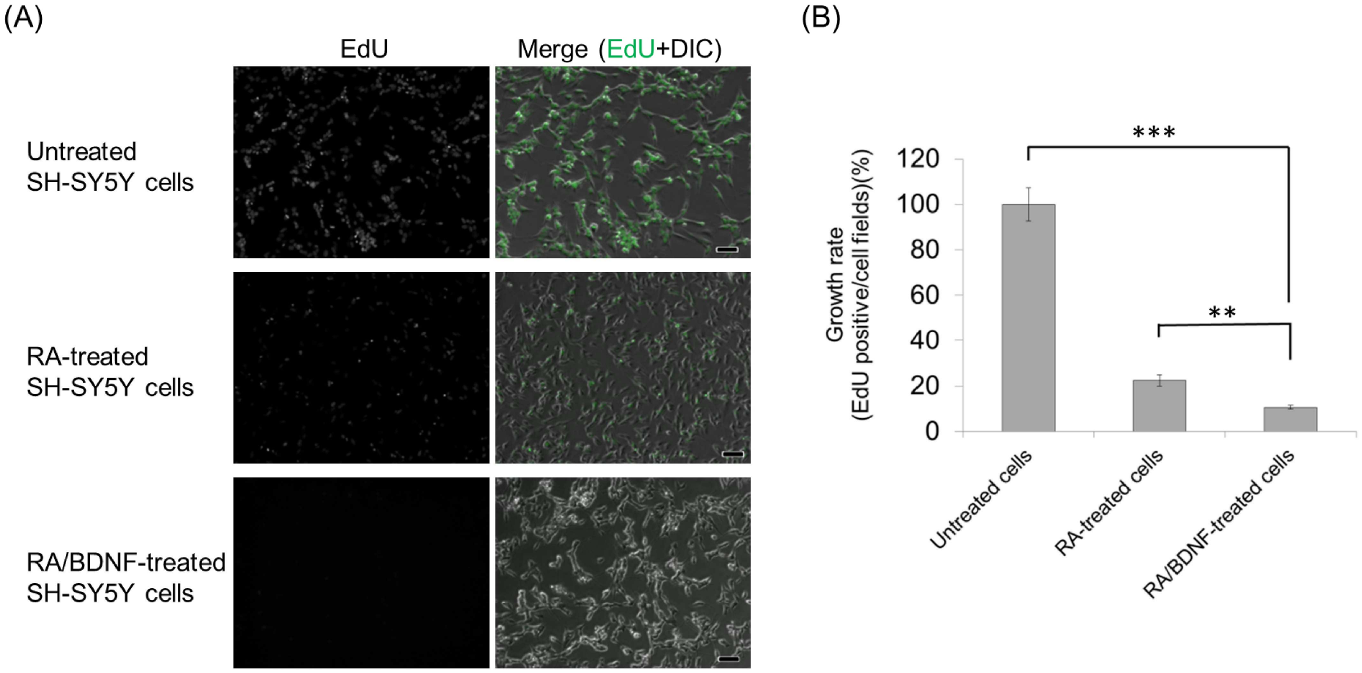

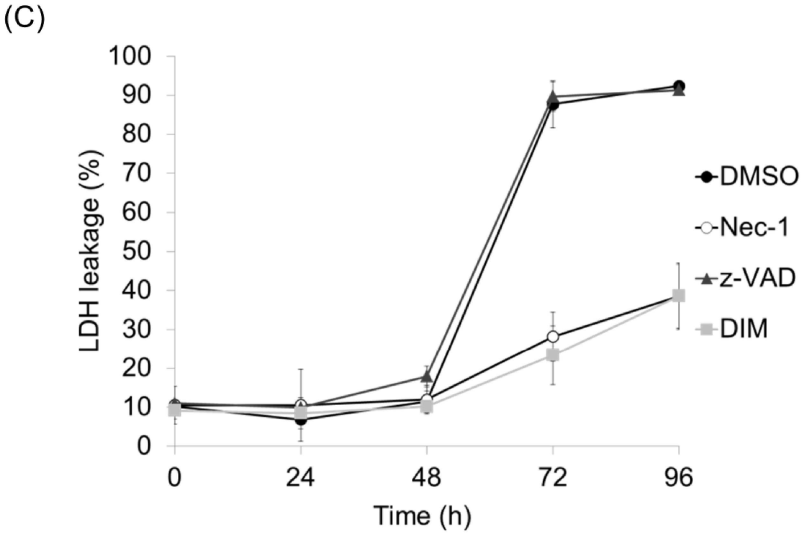

Supplementary Figure 2 Validation of shRNA for silencing RIP3 in Jurkat cells (related to Figure 2).

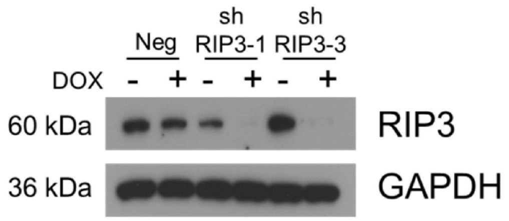

Supplementary Figure 3 MPP+-induced loss of the mitochondrial membrane potential is inhibited by DIM, but not morphological changes of the mitochondria (related to Figure 3).

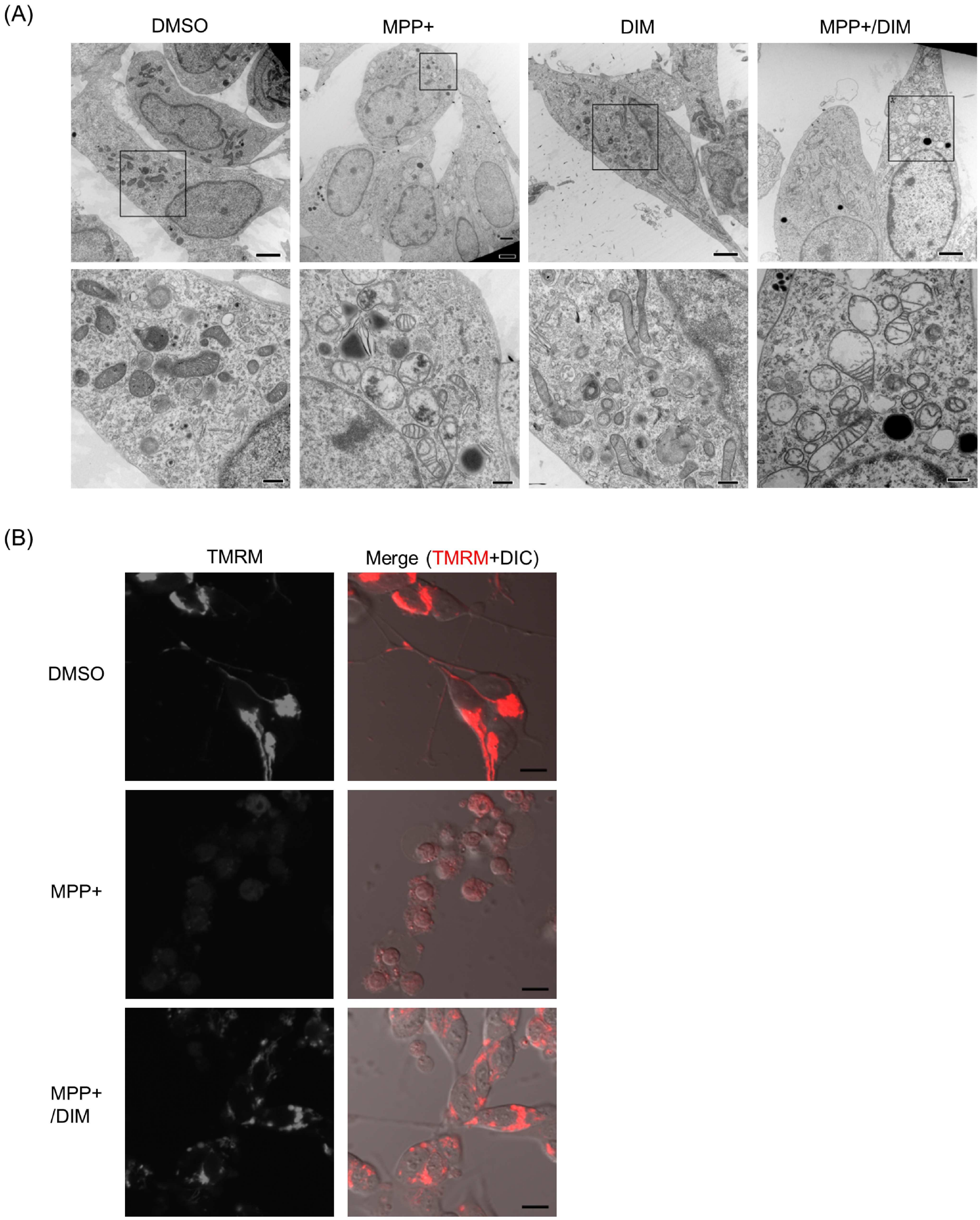

Supplementary Figure 4 p53-deficient SH-SY5Y cells generated by the CRISPR/Cas9 system (related to Figure 4).

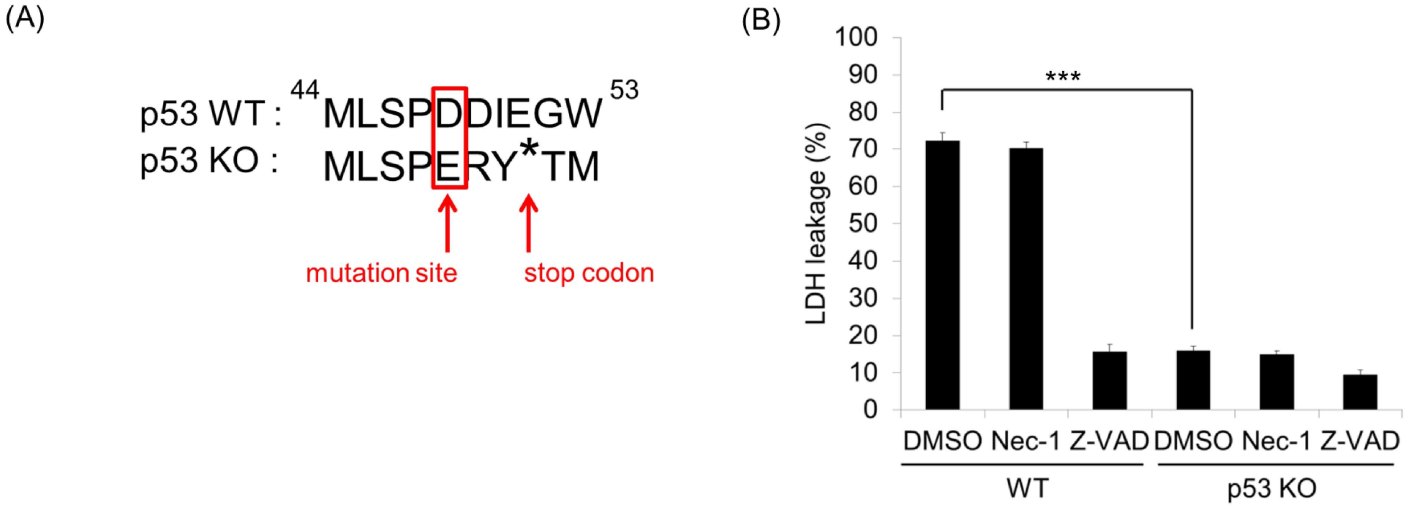

Supplementary Figure 5 Inhibition of erastin- and RSL3-induced ferroptosis by DIM and NAC, but not Nec-1 (related to Figure 5).

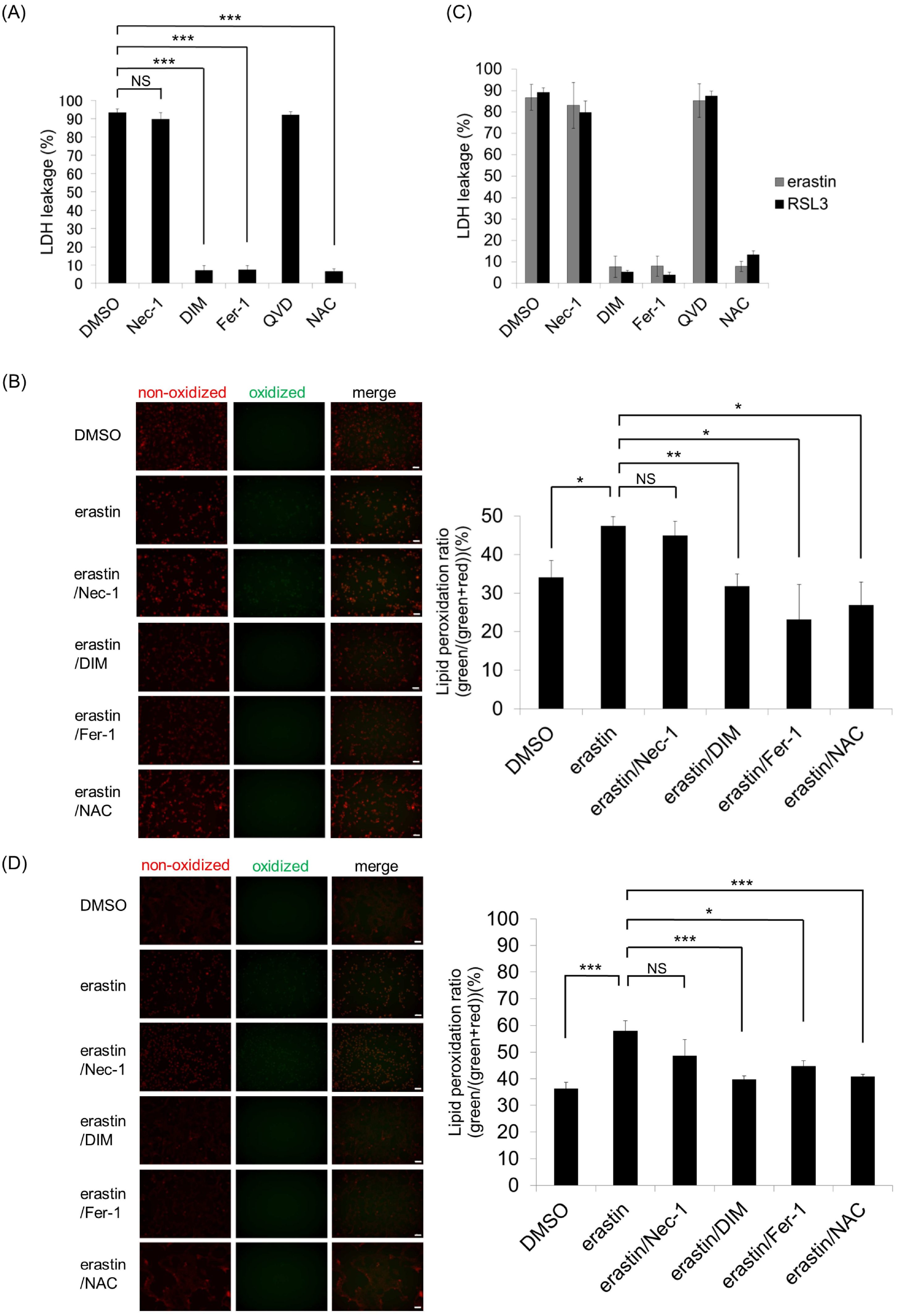

Supplementary Figure 6 Erastin-induced ferroptosis is enhanced by metal ions (related to Figure 6).

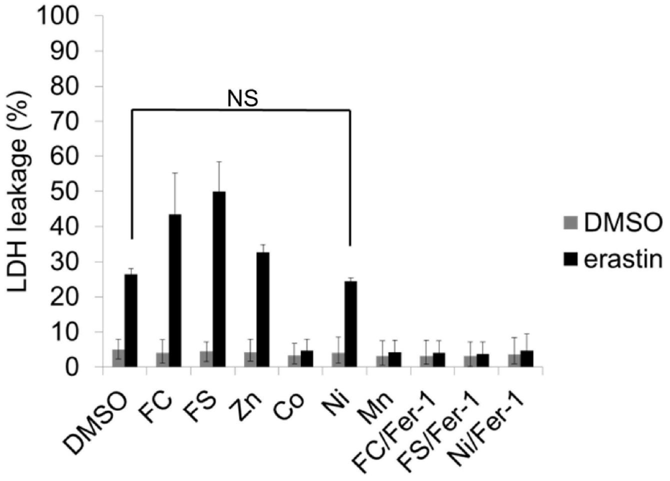

Supplementary Figure 7 MPP+-induced cell death and lipid peroxidation are not inhibited by NAC.

(A)

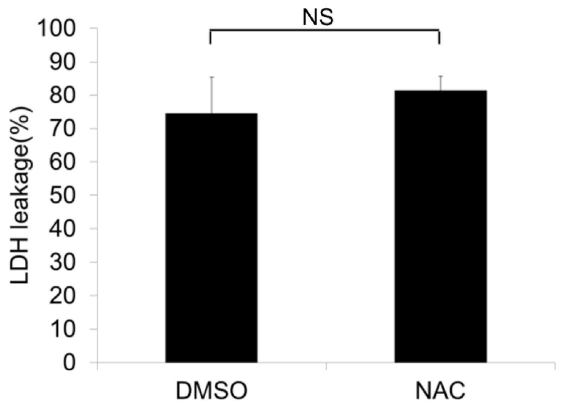

(B)

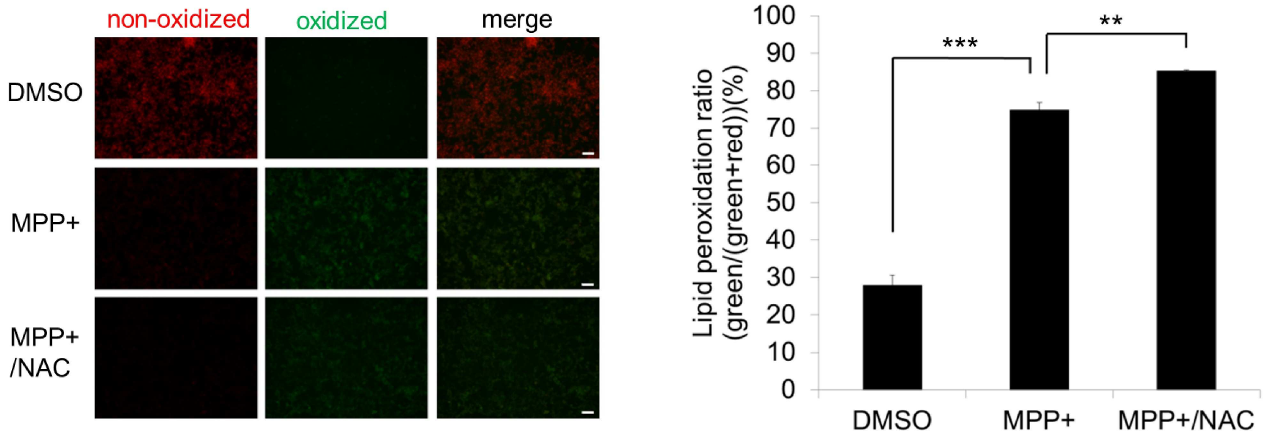

Supplement: Supplementary Figures [file cddiscovery201713-s1.pdf]
